# Hemodynamics in neonates with polycythemia before and after partial exchange transfusion: an observational study

**DOI:** 10.3389/fped.2023.1296184

**Published:** 2024-01-05

**Authors:** Aditya Kallimath, Karthik Kolkur, Nandini Malshe, Jan Klimek, Pradeep Suryawanshi

**Affiliations:** ^1^ Department of Neonatology, Bharati Vidyapeeth Deemed University Medical College, Pune, India; ^2^ Neonatal Intensive Care Unit, Westmead Hospital, Wentworthville, NSW, Australia

**Keywords:** neonate, polycythemia, partial exchange transfusion, hemodynamic parameters, echocardiographic parameters

## Abstract

**Introduction:**

The current recommendations for the management of neonatal polycythemia are that partial exchange transfusion (PET) should be performed if the hematocrit is >70% in an asymptomatic neonate, or if the haematocrit is >65% in a symptomatic neonate. The hemodynamic effects of PET for neonatal polycythemia have not been well researched.

**Objectives:**

To evaluate the hemodynamic effects of PET in neonates with polycythemia.

**Methodology:**

Prospective observational study conducted in a neonatal intensive care unit of a tertiary care teaching hospital enrolling 21 neonates with polycythemia who underwent PET. Hemodynamic and echocardiographic parameters were obtained prior to PET and 6 h after procedure.

**Results:**

The mean gestational age of neonates with polycythemia was 35.08 ± 2.35 weeks with a mean birth weight of 1,929 ± 819.2 g. There was a significant improvement noted in heart rate and oxygen saturation post PET procedure (*p* < 0.05). Right ventricular systolic function parameters showed significant improvement (Tricuspid annular plane systolic excursion, fractional area change, right ventricular output) (*p* < 0.05). Left ventricular function parameters showed significant improvement (Fractional shortening, left ventricular output, E:A ratio) (*p* < 0.05). Resolution of symptoms was noted after PET procedure with no adverse events associated with PET.

**Conclusion:**

PET maybe effective in improving heart rate and oxygen saturation levels in polycythemic neonates. It has good short-term hemodynamic stability with improvement in right ventricular systolic, as well as left ventricular systolic and diastolic function. It is a safe and effective procedure with minimal adverse effects. Further studies with larger sample size and a control group would be required to corroborate our findings.

## Introduction

Neonatal polycythemia is observed in 1–5 percent of neonates (1). Based on published guidelines a venous hematocrit (HCT) of greater than or equal to 65% is defined as neonatal polycythemia (2). Despite the fact that many affected newborns display no symptoms, it is believed that hyperviscosity and/or metabolic consequences of an enlarged red blood cell mass are the causes of distinctive clinical characteristics (1, 2). The incidence of polycythemia may have been influenced by the practice of delayed cord clamping but Cochrane review states that there is no difference in neonatal polycythemia between early and late cord clamping groups (3). Although the pathophysiology of polycythemia is multifactorial, passive (secondary to erythrocyte transfusion) and active (increased intrauterine erythropoiesis) factors are two main contributing categories (4).

Most affected newborns are asymptomatic. If symptomatic, non-specific signs and symptoms may include gastrointestinal signs and symptoms (poor feeding or vomiting), hypoglycaemia, plethora, cyanosis, lethargy, and hypotonia. Severe complications may include respiratory distress, seizures, necrotizing enterocolitis (NEC), and pulmonary hypertension (5). Symptoms, when present, often begin by two hours after birth, after fluid shifts have occurred and the hematocrit is at its peak (5).

All polycythemia infants should be observed closely for neurologic and cardiovascular symptoms and monitored for common complications, such as hypoglycemia and hyperbilirubinemia. Intravenous (IV) hydration and partial exchange transfusion (PET) are two interventions to reduce HCT. Current recommendations are that PET should be performed if the hematocrit is >70% in an asymptomatic neonate, or if the haematocrit is >65% together with the symptomology mentioned above (6).

In a study done by Ergenekon et al. on neonates with polycythemia who underwent PET, it was found that there was an increase in cerebral oxygenation and an improvement in microcirculation as shown by near-infrared spectroscopy (NIRS) (7). In another similar study by Murphy et al, it was noted that polycythemia was associated with relative bradycardia and increased pulmonary vascular resistance (PVR), with a normalization of heart rate and a decrease in pulmonary vascular resistance post PET (8). Although insufficient, currently-available data suggests that PET may result in an earlier improvement of symptoms; however, there is no evidence of long-term benefit from PET (9). The hemodynamic effects of PET for neonatal polycythemia have not been well researched. This study was carried out to evaluate the hemodynamic effects of PET in neonates with polycythemia.

## Material and methods

### Study settings and subjects

This was a single-center prospective observational study conducted in the neonatal intensive care unit (NICU) of a tertiary care teaching hospital. Partial exchange transfusion was performed on polycythaemic neonates who (a) were asymptomatic with a venous hematocrit of more than 70%, or (b) were symptomatic with venous hematocrit of more than 65%. Symptoms included respiratory distress, cyanosis, poor perfusion (capillary refill time more than 3 s), lethargy, feed intolerance, apnea, hypoglycemia (less than 47 mg/dl in first 48 h of life and less than 60 mg/dl after 48 h of life), and seizures.

Any neonates with polycythemia who underwent PET and required inotropes (before or within 6 h after the procedure) or were diagnosed with sepsis were excluded from the study. The study was commenced after approval from the institutional ethics committee and informed parental consent was obtained prior to the study. A baseline clinical and hemodynamic status was recorded and entered in a pre-designed case record form before PET.

Partial exchange transfusion (PET) was performed via stable vascular access. Hemodynamic parameters included heart rate (HR), noninvasive blood pressure (NIBP), preductal peripheral oxygen saturation (SpO2), and echocardiographic evaluation. The amount of blood to be removed was calculated based on the formula: Blood to be exchanged (mL) = (actual HCT−desired HCT) × blood volume per kilogram/actual HCT.

A nomogram designed for clinical use by Rawlings et al, correlating blood volume per kilogram with birth weight in polycythemia neonates, was used for blood volume calculation (10). Blood was drawn in 5 ml aliquots and replaced with an equal amount of normal saline. Hemodynamic parameters (HR, NIBP, SpO2) and echocardiographic evaluation were assessed again after 6 h of the PET procedure.

Neonatologists trained in functional echocardiography obtained both the right and left ventricular systolic and diastolic function measurements. Right ventricular measurements included: tricuspid annular plane systolic excursion (TAPSE), right ventricular output (RVO), fractional area change (FAC), and E: A ratio. Left ventricular measurements included: left ventricular output (LVO), fractional shortening (FS), and E: A ratio. Pulmonary pressures were estimated using peak velocity of tricuspid regurgitation (11). Measurements were done using a Philips 50G machine, with 12-4 MHz high-frequency phased array transducer probes, and using published methods. Functional echocardiography was done before the PET procedure and again 6 h after completion of the PET. Each of the functional echo parameters was repeated twice for reproducibility, and to reduce errors. The outcome measured was the change in hemodynamic parameters of the neonate 6 h after the PET was done.

### Statistical analysis

The Statistical analysis was performed by SPSS 23.0 version. Categorical variables were described by taking percentages. Continuous variables were described as mean and variation of each observation from the mean value (Standard deviation) represented as mean ± SD. Continuous Paired data was analyzed using Paired *T*-test. Variables with *p*-value < 0.05 were considered statistically significant.

## Results

**Figure 1:**
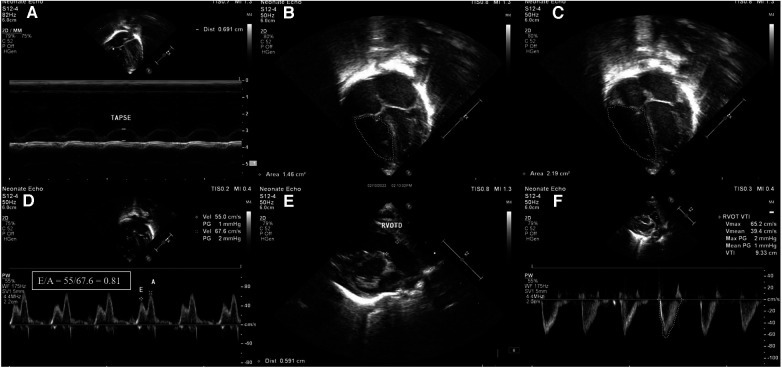
Images of right ventricular systolic and diastolic function parameters in echocardiography. (**A**) Tricuspid annular plane systolic excursion (TAPSE) shown in M-mode. (**B**) Fractional area change (FAC) in apical 4 chamber view-end systolic area (1.46 cm^2^). (**C**) Fractional area change (FAC) in apical 4 chamber view-end diastolic area (2.19 cm^2^). (**D**) Apical 4 chamber view pulse wave Doppler across tricuspid valve showing E/A ratio of 0.81. (**E**) Measurement of RVOT diameter (RVOTD) in parasternal long axis view (PLAX). (**F**) Right ventricular velocity time integral measured in short-axis view using pulse wave Doppler (VTI). Stroke volume = cross-sectional area × velocity time integral.

**Figure 2:**
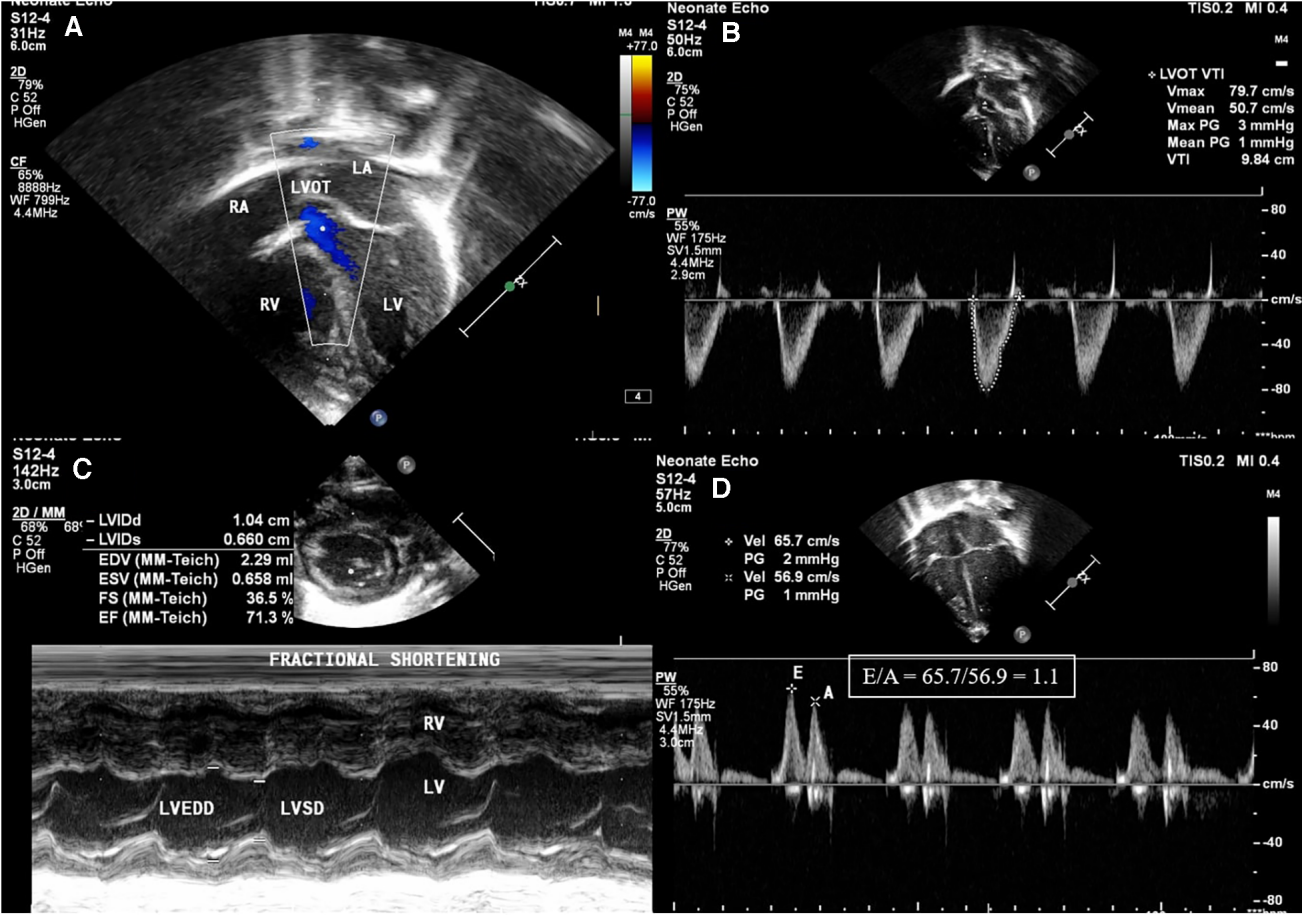
Images of left ventricular systolic and diastolic function parameters in echocardiography. (**A**) Left ventricular outflow tract (LVOT) seen in apical 5 chamber view. (**B**) Left ventricular velocity time integral measured in apical view with LVOT using pulse wave Doppler (VTI): shown in the figure as 9.84 cm. (**C**) Fractional shortening (FS) in the parasternal short axis (PSAX) view using M-mode using equation FS = LVEDD-LVESD/LVEDD. (**D**) Apical 4 chamber view pulse wave Doppler across mitral valve showing E/A ratio of 1.1.

There were a total of 1,758 NICU admissions during the study period, from July 2022 to July 2023. Of the 48 neonates who were diagnosed with polycythemia, 32 were symptomatic and all 32 were managed with PET. Of the 32 neonates who underwent PET, 21 were included in the study, and 7 were excluded because of being diagnosed to have sepsis or because they required inotropic support. The remaining 4 neonates were not included as an echocardiographic assessment could not be performed on them at the time. The study group (*n* = 21) comprised 5 term and 16 preterm neonates (mean gestational age of 35.08, SD ± 2.35 weeks) with a mean birth weight of 1,929 (SD ± 819.2) g. The maternal and neonatal characteristics are elicited in Table 1. The mean age at which polycythemia was diagnosed was [mean (SD)] 20.8 (21.7) h of life with a mean venous hematocrit of 68.8 (SD ± 3.6) and the mean age at which PET was performed was [mean (SD)] 24.33 (23.31) h of life. There was a significant increase noted in heart rate from a baseline mean of 108.9 (SD ± 3.4) to 125.4 beats per minute (SD ± 4.3, *p* < 0.001) 6 h after the procedure. Oxygen saturation (SpO2) showed a significant improvement from a mean baseline of 93.2% (SD + 1.4) to 94.4% (SD + 1, *p* = 0.002) (Table 2). In the right ventricular function parameters, significant changes were noted in TAPSE (from baseline 8.13 mm to 9.19 mm, *p* = 0.001), FAC (from baseline 32% to 36.8%, *p* < 0.001), and RVO (from baseline 181.19 ml/kg/min to 227.24 ml/kg/min, *p* = 0.001) (Figure 1). In the left ventricular function parameters, significant changes were noted in FS (from baseline 36.8% to 39.6%, *p* = 0.02), LVO (From baseline 140.76 ml/kg/min to 186.24 ml/kg/min), and E: A ratio (0.86 to 0.93, *p* = 0.005) (Table 3) (Figure 2). Only 1 neonate (4.7%) was found, prior to PET, to have high pulmonary pressures suggestive of persistent pulmonary hypertension of the newborn (PPHN) with pressures of 45 mm of Hg; there was a decrease in pressures to below 30 mm of Hg after the PET procedure.

**Table 1:** Maternal and neonatal characteristics of the cohort.

Maternal characteristics	
Primigravida, *n* (%)	11 (52.3%)
Gestational diabetes maellitus, *n* (%)	02 (9.5%)
Hypertension disorder, *n* (%)	06 (28.6%)
Doppler abnormalities, *n* (%)	04 (19%)
Intrauterine growth restriction, *n* (%)	03 (14.3%)
Twin gestation, *n* (%)	04 (19%)
Mode of delivery lower section cesarean section, *n* (%)	16 (76.2%)
Neonatal characteristics	
Gestational age (Mean ± SD)	35.08 ± 2.35
Birth weight (grams) (Mean ± SD)	1,929 ± 819
Delayed cord clamping was done, *n* (%)	07 (33.3%)
APGAR at 1 min (Mean ± SD)	7.62 ± 0.49
APGAR at 5 min (Mean ± SD)	8.86 ± 0.36
Clinical findings (*n* = 21) Respiratory distress Cyanosis Hypoglycemia Lethargy Feed intolerance	19 (90.5%) 07 (33.3%) 05 (23.8%) 05 (23.8%) 01 (4.7%)
Polycythemia detected at the hour of life (Mean ± SD)	20.38 ± 21.17
Partial exchange transfusion done at the hour of life (Mean ± SD)	24.33 ± 23.31

**Table 2:** Vital parameters of the cohort before and 6 hours after partial exchange transfusion.

Vital parameters	Before PET	After PET	*p*-value
Heart rate (beats per minute), mean (SD)	108.95 ± 3.44	125.43 ± 4.34	**<0** **.** **001**
Systolic blood pressure (mm of Hg), mean (SD)	54.35 ± 3.6	55.71 ± 2.57	0.193
Diastolic blood pressure (mm of Hg), mean (SD)	36.12 ± 3.6	37.12 ± 2.42	0.161
Mean blood pressure (mm of Hg), mean (SD)	42.24 ± 2.99	45.29 ± 5.73	0.064
Oxygen saturation (SpO2%), mean (SD)	93.24 ± 1.48	94.48 ± 1.08	**0**.**002**

Values in bold indicate statistical significance.

**Table 3:** Echocardiographic parameters of the cohort before and 6 h after partial exchange transfusion.

Variable	Parameters before PET	Parameters 6 h after PET	*p*-value	Normal reference range
Left ventricular function parameters				
Fractional shortening (%), mean (SD)	36.85 ± 4.15	39.65 ± 4.52	**0** **.** **02**	27%−42%
Left ventricular output ml/kg/min, mean (SD)	140.76 ± 37.39	186.24 ± 44.89	**<0**.**001**	150–300 ml/kg/min
E/A ratio, mean (SD)	0.97 ± 0.1	1.01 ± 0.09	**0**.**005**	1
Right ventricular function parameters				
Tricuspid annular plane systolic excursion (mm), mean (SD)	8.13 ± 1.51	9.19 ± 1.6	**0**.**001**	8–12 mm
Fractional area change (%), mean (SD)	32.05 ± 4.55	36.8 ± 2.75	**<0**.**001**	25%−45%
Right ventricular output ml/kg/min, mean (SD)	181.19 ± 71.14	227.24 ± 61.66	**0**.**001**	150–300 ml/kg/min
E/A ratio, mean (SD)	0.86 ± 0.23	0.93 ± 0.12	0.157	1
Pulmonary pressures measured by peak velocity across tricuspid regurgitation jet				
Pulmonary artery systolic pressure, mm of Hg, mean (SD)	24.05 ± 7.34	23.19 ± 5.8	0.392	<35

Values in bold indicate statistical significance.

19 neonates required ventilatory support, of which 5 neonates required invasive ventilatory support. The mean duration of invasive ventilation days was 1.4 ± 0.55 days and the mean duration of noninvasive ventilation was 2.37 ± 1.3 days.

Following the PET procedure, improvement in symptomology was noted in the form of decreased need for ventilatory support, improved perfusion and activity, and euglycemic control. None of the neonates developed any complications such as sepsis, hypotension, or necrotizing enterocolitis (NEC) following PET procedure.

## Discussion

In neonates with polycythemia, when venous HCT is greater than 65%, a small increase in HCT may lead to hyperviscosity (12). Hyperviscosity is said to lead to cardiorespiratory instability due to increased resistance to blood flow in vessels, increased systemic vascular resistance (SVR) and pulmonary vascular resistance (PVR), and a decrease in cardiac output (13). Isovolumetric PET reduces the HCT and thereby viscosity and does not lead to hypovolemia.

Our study aimed to demonstrate the hemodynamic effects of PET in neonatal polycythemia and to study the efficacy of the treatment with improvement in systemic blood flow and oxygen transport. In our study, the polycythemic neonates were in relative bradycardia before PET [(108.9 (SD ± 3.4) compared to 125.4 beats per minute (SD ± 4.3, *p* < 0.001)] post PET, which is similar to the study done by Murphy et al. (116 ± 13–125 ± 16, *p* < 0.05) and Swetnam et al, where relative bradycardia was present in neonates with polycythemia before PET (127 + 7.5–139 + 7.8, *p* < 0.05) (8, 14). We may postulate from these studies that neonates with polycythemia are in a state of relative bradycardia and resolution of polycythemia may increase the baseline heart rate. There was a clinical resolution of cyanosis as there was a significant improvement in oxygen saturation (SpO2) and peripheral circulation post-PET.

Partial exchange transfusion led to an increase in cerebral blood flow and cerebral oxygenation with a decrease in cerebral fractional tissue oxygen extraction (CFTOE) and peripheral microcirculation with evaluation by NIRS in a study by Ergenekon et al. (7).

Polycythemia is known to cause an increase in SVR leading to increased afterload and also affect myocardial function due to the same reason; PET can lead to a drop in afterload, leading to improved ventricular function. This was found in our study where we demonstrated a significant improvement in left ventricular systolic and diastolic function along with improved LVO. There was also a clinically significant increase in the heart rate from the relative bradycardia. We also noted an improvement in blood pressure, although this was not clinically significant; an improvement in BP was previously noted in a single case report by Sehgal et al. (15). Similar to our study, improvement in indices including the heart rate, left ventricular and right ventricular systolic time interval, and stroke volume index, was noted in a study evaluating 13 infants with polycythemia who underwent PET by Swetnam et al. (14). The other significant finding noted in our study was the improvement noted in right ventricular systolic function and RVO. This again can be attributed to a reduction in PVR, also found in the study by Swetnam et al. (14).

It has to be noted though that in normal neonates, during the early postnatal period, there is improvement in echocardiographic findings within the first 72 h. A study by Ha KS et al. had shown significant changes in systolic, diastolic, and tissue doppler imaging parameters, after 24 h of life (16).

Polycythemia is also known to cause persistent pulmonary hypertension of the newborn (PPHN) by its effect on increasing PVR (17), but in our study, only one neonate (4.7%) was diagnosed to have PPHN before the PET. Neonates who were polycythemic and underwent PET and who also received milrinone for PPHN were excluded from our study; this could explain the low incidence of PPHN in our study.

Ozek et al. published a Cochrane review on PET which showed a slightly increased risk of NEC in infants who had undergone the procedure (18). In our study, none of the neonates had developed NEC after the procedure, nor were there any issues of feed intolerance following the procedure.

This prospective cohort study shows that isovolumetric PET is a safe procedure leading to acute hemodynamic benefits with improvement in right ventricular systolic function, as well as left ventricular systolic and diastolic function.

This is one of the very few studies describing the effect of PET on clinical, hemodynamic, and echocardiographic parameters in neonates with polycythemia. The study population included both term and preterm neonates. The limitations of our study would include a small sample size of only 21 neonates from a single center with no sampling calculation before starting the study, blinding not performed during our study, no control group, and no long-term follow-up of our cohort. It is also difficult to definitively assert our findings due to a small sample size, and it will be necessary to carry out an additional study with a larger sample to confirm them.

## Conclusion

Partial exchange transfusion may be effective in improving heart rate and oxygen saturation levels in polycythemic neonates. It has good short-term hemodynamic stability with improvement in right ventricular systolic, as well as left ventricular systolic and diastolic function. It is a safe and effective procedure with minimal adverse effects. Further studies with a larger sample size and a control group would be required to corroborate our findings.

## Data Availability

The raw data supporting the conclusions of this article will be made available by the authors, without undue reservation.
